# Ageism directed to older adults in health services: A scoping review

**DOI:** 10.1590/1518-8345.6727.4020

**Published:** 2023-10-06

**Authors:** Pricila Oliveira de Araújo, Isabela Machado Sampaio Costa Soares, Paulo Roberto Lima Falcão do Vale, Anderson Reis de Sousa, Elena Casado Aparicio, Evanilda Souza de Santana Carvalho

**Affiliations:** 1 Universidade Estadual de Feira de Santana, Feira de Santana, BA, Brasil.; 2 Ministério da Saúde, Brasília, DF, Brasil.; 3 Universidade Federal do Recôncavo da Bahia, Santo Antônio de Jesus, BA, Brasil.; 4 Universidade Federal da Bahia, Escola de Enfermagem, Salvador, BA, Brasil.; 5 Universidad Complutense de Madrid, Madrid, España.

**Keywords:** Ageism, Aged, Health Services, Aging, Health Personnel, Review, Ageísmo, Anciano, Servicios de Salud, Envejecimiento, Personal de Salud, Revisión, Etarismo, Idoso, Serviços de Saúde, Envelhecimento, Pessoal da Saúde, Revisão

## Abstract

**Objective:**

to map the expressions of ageism directed to older adults in health services and the respective coping measures.

**Method:**

a scoping review of primary studies in English, Spanish and Portuguese, without time delimitation and collected from 14 databases. Selection of the titles, abstracts and full text was in charge of two independent and blinded reviewers, totaling a *corpus* comprised by 41 articles. Data extraction was performed by pairs. The data were presented in narrative summaries and charts.

**Results:**

the ageism expressions are understood at the interpersonal level through images and attitudes that depreciate, devalue life and delegitimize older adults’ needs, as well as at the institutional level, which confers barriers to accessing health services, generating non-assistance and neglect. The coping measures consist of educational interventions and expansion of communication channels between aged people, health professionals and managers.

**Conclusion:**

the results may make health professionals vigilant for care/neglect guided by age bias and sensitive for coping with ageism by obtaining diverse scientific knowledge. The analysis of the phenomenon in the Unified Health System context constitutes a knowledge gap, as well as the implicit ageism expressions.

## Abstract (es)

**Objetivo:**

mapear las expresiones y medidas para combatir el edadismo contra las personas mayores en los servicios de salud.

**Método:**

revisión de alcance de estudios primarios en inglés, español y portugués, sin delimitación temporal, recopilados de 14 bases de datos. Dos revisores independientes realizaron de forma ciega la selección de títulos, resúmenes y texto completo, el *corpus* estuvo compuesto por 41 artículos. La extracción de datos se realizó por pares. Los datos se presentaron en resúmenes narrativos y cuadros.

**Resultados:**

las expresiones de edadismo se manifiestan a nivel interpersonal, mediante imágenes y actitudes que deprecian, infravaloran la vida y deslegitiman las necesidades de las personas mayores, y a nivel institucional, por medio de barreras para acceder a los servicios de salud, que provocan falta de atención y descuido. Las medidas para combatirlo consisten en intervenciones educativas y ampliación de los canales de comunicación entre las personas mayores, los profesionales de la salud y gestores.

**Conclusión:**

los resultados pueden hacer que los profesionales de la salud estén atentos a la (falta de) atención debido al sesgo etario y sensibilizarlos para que enfrenten el edadismo mediante la adquisición del conocimiento científico. Hay lagunas de conocimiento tanto en el análisis del fenómeno en el contexto del Sistema Único de Salud como sobre las expresiones implícitas de edadismo.

## Abstract (pt)

**Objetivo:**

mapear as expressões e medidas de enfrentamento do ageísmo direcionado às pessoas idosas em serviços de saúde.

**Método:**

revisão de escopo de estudos primários em inglês, espanhol e português, sem delimitação temporal, coletados em 14 bases de dados. A seleção dos títulos, resumos e texto completo foi realizada por dois revisores independentes e às cegas, totalizando *corpus* de 41 artigos. A extração dos dados foi realizada por pares. Os dados foram apresentados em resumos narrativos e quadros.

**Resultados:**

expressões de ageísmo estão compreendidas no nível interpessoal, por meio de imagens e atitudes que depreciam, desvalorizam a vida e deslegitimam as necessidades das pessoas idosas, e o nível institucional, que confere barreiras ao acesso dos serviços de saúde, gerando desassistência e descuidado. As medidas de enfrentamento consistem em intervenções educativas e ampliação dos canais de comunicação entre pessoas idosas, profissionais da saúde e gestores.

**Conclusão:**

os resultados poderão tornar os profissionais da saúde vigilantes para o (des)cuidado guiado pelo viés de idade e sensíveis para o enfrentamento do ageísmo pela obtenção de conhecimentos científicos. A análise do fenômeno no contexto do Sistema Único da Saúde constitui lacuna do conhecimento, bem como as expressões implícitas do ageísmo.

**Highlights:**

(1) Ageism expressions involve interpersonal and institutional relationships.

(2) Ageism directed to older adults permeates from diagnosis to treatment.

(3) The coping measures comprise educational actions and also scientific research studies.

(4) It is necessary to recognize the presence of ageism in health services.

(5) It is necessary to recognize care/neglect practices guided by age bias.

## Introduction

The term “ageism” was first used to refer to disquiet, revulsion and aversion on the part of young and middle-aged people towards aging, illness, disability, impotence, “uselessness” and death, and is usually linked to people because of their age( 1 ). Currently, it is recognized that ageism can be directed at any age group; however, up to the present day, the focus on older adults has received more attention since, in Western contexts, it is common for them to be represented as frail, weak, dependent, non-productive and whose health problems are naturalized and understood as a normal part of aging( 2 ).

The literature treats ageism as a multifaceted concept, which involves three different dimensions: cognitive (stereotypes); affective (prejudice); and behavioral (discrimination). The cognitive dimension encompasses negative stereotypes about aging. They are acquired very early in life and tend to act as self-fulfilling prophecies in old age( 3 ) — for example, thinking that old people are incapable of learning new things. Stereotypes are activated when there is certain disregard for the specificities of aged people; they can generate labels that mean separating people into different categories and activating beliefs that depreciate individuals and cause negative consequences in different life areas( 4 ).

The affective dimension (prejudices) consists of an emotional reaction or negative or positive feelings that create differences in groups or outside them. For example, when a person feels sorry for older adults for considering them frail, which motivates disregarding their ability to do something alone; and behavioral, which comprises the discrimination that occurs when exclusionary practices are used towards third parties and these individuals are placed in unfavorable social positions due to age; for example, when an aged worker is prohibited from attending a training session on account of age( 1 ). Ageism can be subtle and hardly noticed or explicit and well known, shaping older people’s perception of their abilities and needs, as well as the view of those around them( 2 ).

With the COVID-19 pandemic, previously veiled discussions became explicit, such as aged people’s social place and the manifestation of ageism expressions by different sectors of society. In this scenario, aged people were portrayed as a social and family burden, frail, stubborn, disobedient and whose lives are devalued and considered less important than that of young people, which in turn causes implications older adults’ mental, emotional and physical health( 5 ).

In the context of health services, the effects of stereotypes, prejudices and discrimination experienced by aged people are well-known and restrict access to health care, diagnoses and treatments, in addition to being significantly associated with worse health conditions, indicating reduced longevity, low quality of life and well-being, health risk behaviors, poor social relationships, physical illness, mental ailments and cognitive impairment( 4 ).

Furthermore, ageism is expressed in the health field through biased and veiled attitudes and practices related to age, which favor younger people over older people in the use of health resources and services, such as access to beds in Intensive Care Units, high-cost treatments and surgical interventions, among others. It is present at the cultural level of Western societies and, at the institutional level, it refers to laws, rules, social norms, policies and protocols that restrict aged people’s opportunities with important repercussions on the health care provided to this population group, in addition to potentiating inequalities in health systems and services( 6 ).

Furthermore, ageism is less studied than other forms of discrimination, with few studies that explicitly examine its manifestations in the health field( 7 ); in addition, there is scarce scientific evidence that supports health professionals’ work process to mitigate the impacts of ageism in old age( 8 ). That said, this review differs from what has already been produced by presenting an expanded overview of how ageism manifests itself in health services.

Mapping the diverse evidence of ageism in health services and the respective coping strategies becomes relevant due to the possibility of providing subsidies for future studies, contributing to the formulation of policies and implementing strategies to reduce this phenomenon in the health field, in addition to helping train health professionals.

A preliminary survey was carried out in February 2021 with the *Medical Subject Headings* (MesH) terms “ageism” and “health”, in the Prospero, PubMed, Open Science Framework, Joanna Briggs Institute Evidence Synthesis and Cochrane Database of Systematic Reviews portals, not finding scoping review studies on the topic. Thus, this study aims at mapping the expressions of ageism directed to older adults in health services and the respective coping measures.

## Method

### Type of study

This is a scoping review conducted in accordance with the methodology proposed by the Joanna Briggs Institute (JBI) for scoping reviews( 9 ), presented according to the recommendations set forth in the Preferred Reporting Items for Systematic reviews and Meta-Analyses extension for Scoping Reviews (PRISMA-ScR): Checklist and Explanation( 10 ) and with its protocol registered on the Open Science Framework platform for the registration of scientific papers via the following link: https://osf.io/pv2by and duly published( 11 ). The research protocol was outlined guided by the research question, which was formulated based on the PCC mnemonic rule, where Population (P) consisted of aged people and health professionals; Concept (C) was ageism directed to older adults; and Context (C) corresponded to health services, which involve all the health system components provided in the primary, secondary and tertiary care levels. Thus, the research questions are as follows: How is ageism directed towards older adults expressed in health services? Which are the coping measures against ageism directed to older adults in health services?

### Study scenarios

This review was performed on the following databases: PubMed via the *National Library of Medicine*/The National Center for Biotechnology Information (NLM/NCBI); Digital library from Scientific Electronic Library Online (SciELO); *Literatura Latino-Americana e do Caribe em Ciências da Saúde* (LILACS), Cumulative Index to Nursing and Allied Health Literature (CINAHL) via Business Source Complete (EBSCO) and PsycINFO, Angeline, Embase, Scopus and Web of Science Core Collection. Choice of the portals and databases was due to their large collections of publications in the areas of the intended study.

The Gray Literature search was conducted through the following portals: *Teses CAPES*; Cybertesis *Digitalis Thesis* Repository; Digital Access to Research Theses - Europe (DART-E) E-theses; *Repositórios Científicos de Acesso Aberto de Portugal* (RCAAP); and Online Knowledge Library.

### Period

Data collection took place between April and May 2021.

### Population

The study population consisted of 41 scientific articles found in searches carried out in the databases and in the gray literature.

### Selection criteria

The evidence sources were the full texts of primary studies (quantitative or qualitative), theses and dissertations, published in the main databases in the Health, Psychology and Gerontology areas, in Portuguese, English and Spanish and without time limits, with the justification of retrieving as many publications as possible. Review studies, opinion articles, theoretical essays, comments, books and book chapters were not included.

### Data collection instrument

An instrument for data extraction organized in a Word (2013) file was prepared, developed by the JBI( 9 ) and adapted to meet the objectives of this review, tested by the research team that and dealing with diverse information regarding identification of the studies: authors, title, year of publication, Digital Object Identifier (DOI) link, journal, publication date, objectives, methodology (type of study, participants, context, concept, research instruments) and results (expressions of ageism and respective coping measures).

### Data collection

An exploratory search was performed in February 2021 in PubMed and CINAHL via EBSCO to identify articles on the subject matter. The keywords found in the titles, abstracts and MeSH descriptors found were selected to comprise the search strategies developed with the support of a librarian specialized in reviews and applied in the databases selected as scenarios for this study (Figure 1).

**Figure 1 -:** Research strategies. Feira de Santana, BA, Brazil, 2021

Information source	Search strategy
PubMed	((“Ageism”[MeSH Terms] OR “Age Discrimination” [All Fields] OR “Age discriminations”[All Fields] OR (“Ageism”[MeSH Terms] OR “Ageism” [All Fields])) AND (“Aged” [MeSH Terms] OR (“Aged” [MeSH Terms] OR “Aged” [All Fields]) OR (“older” [All Fields] OR “olders” [All Fields]) OR “elder*”[All Fields] OR (“senior” [All Fields] OR “seniorities” [All Fields] OR “seniority” [All Fields] OR “seniors” [All Fields]) OR “geriatr*”[All Fields]) AND (“Health”[MeSH Terms] OR (“Health”[MeSH Terms] OR “Health”[All Fields] OR “healths”[All Fields] OR “healthful”[All Fields] OR “healthfulness” [All Fields] OR “healths” [All Fields]))) Filters: Aged 65+ years old, 80 and over: 80+ years old, Middle-Aged: 45-64 years old.
CINAHL*	MH “ageism” OR TI ( ageism or “age discrimination” or “age bias” or “age stereotype” OR “age prejudice” ) OR AB ( ageism or “age discrimination” or “age bias” or “age stereotype” OR “age prejudice”) AND MH aged OR TI ( aged OR elderly OR senior OR “older people” OR geriatric OR elder* ) OR AB ( aged or elderly or senior or “older people” or geriatric OR elder* ) AND MH Health OR TI ( health OR “health services” OR “Health Care Services” OR “Public Health Service” ) OR AB ( health OR “health services” OR “Health Care Services” OR “Public Health Service” ) AND (Restrict by SubjectAge: - aged, 80 & over Restrict by SubjectAge: - middle-aged: 45-64 years old Restrict by SubjectAge: - aged: 65+ years old) AND (Restrict by academic journals)

*CINAHL = Cumulative Index to Nursing and Allied Health Literature

All the studies identified were grouped and sent to the EndNote tool (Clarivate Analytics, United States of America) to remove duplicates. Subsequently, independently and blindly and using the Rayyan Intelligent Systematic Review tool (https://www.rayyan.ai/), two reviewers selected the evidence sources by reading and selecting titles, abstracts and full texts; any and all disagreements were solved by a third reviewer, and the reasons for excluding productions were quantified and justified. Data extraction was performed by two reviewers, one for data collection and the other for data review and confirmation.

Finally, the reference lists of the articles retrieved in the full-text search and included in the final selection were screened for inclusion in the study sample. The authors of scientific productions whose full texts were unavailable in the databases were contacted; however, access to them was not granted.

### Data treatment and analysis

The data extracted were organized in the MaxQDA software, which is part of the QDA family (Qualitative Data Analysis Software), 2020 version, and analyzed based on simple descriptive statistics, presented in narrative summaries and charts and discussed in the light of other national and international findings on the topic.

### Ethical aspects

The studies used offer public domain access, waiving the need to submit the study to the Research Ethics Committee.

## Results

**Figure 2 -:**
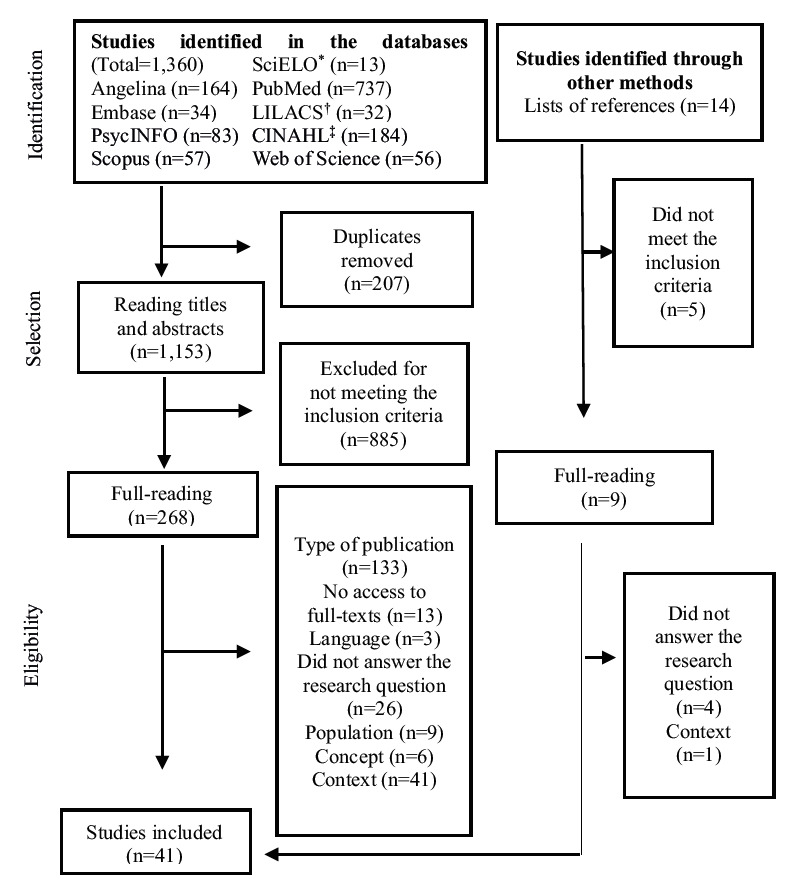
Flowchart corresponding to the selection process of primary studies included in the scoping review. Feira de Santana, BA, Brazil, 2021

^*^SciELO = Digital Library from Scientific Electronic Library Online; †LILACS = *Literatura Latino-Americana e do Caribe em Ciências da Saúde*; ‡CINAHL = Cumulative Index to Nursing and Allied Health Literature

The final sample of the review consisted of 41 scientific articles, which are presented in the PRISMA-SrC flowchart( 12 ) in Figure 2.

The characterization of the scientific production on ageism directed to older adults in health services is presented in Figure 3.

**Figure 3 -:** Characterization of the scientific production on ageism directed to older adults in health services. Feira de Santana, BA, Brazil, 2021

Reference	Authors	Title	Year	Objectives	Methods	Language
( 13 )	Grant PT, Henry JM, McNaughton GW.	The management of elderly blunt trauma victims in Scotland: evidence of ageism?	2000	To determine the in-hospital mortality of traumatized aged patients. To analyze the main characteristics of its management; to see whether these traumatized patients were treated less aggressively than their younger peers.	A prospective study with data collected by Scottish Trauma Audit Group, in Scotland.	English
( 14 )	Uncapher H, Areán PA.	Physicians are less willing to treat suicidal ideation in older patients.	2000	To determine whether there is age bias among primary care physicians when contemplating the treatment of suicidal patients.	A quantitative study with primary care physicians in San Francisco, United States.	English
( 15 )	Bouman WP, Arcelus J.	Are psychiatrists guilty of “ageism” when it comes to taking a sexual history?	2001	To determine the current perceived practice of consulting psychiatrists regarding their patients’ sexual history and management of sexual dysfunction.	A quantitative study with aged people and psychiatrists in hospitals from Sheffield, Nottingham, Leicester and Birmingham, England.	English
( 16 )	Kennelly C, Bowling A.	Suffering in deference: a focus group study of older cardiac patients’ preferences for treatment and perceptions of risk.	2001	To explore older adults’ health care experiences in relation to their medical condition (ischemic heart disease), their understanding of health risks, treatment preferences, and the impact of the treatments on their quality of life.	A qualitative study carried out in London with people over 56 years of age and diagnosed with ischemic heart disease.	English
( 17 )	Austin D, Russell EM.	Is There Ageism in Oncology?	2003	To determine whether age-related differences in hospital management of cancer patients were or were not clinically justifiable.	A quantitative study with cancer patients in a colorectal cancer treatment hospital from Scotland.	English
( 18 )	Fischer LR, Wei F, Solberg LI, Rush WA, Heinrich RL.	Treatment of elderly and other adult patients for depression in primary care*.*	2003	To determine whether depression is treated differently in older and younger patients in primary care clinics.	A quantitative study with adults diagnosed with depression treated at primary care clinics in the Midwest Region of the United States.	English
( 19 )	Peake MD, Thompson S, Lowe D, Pearson MG.	Ageism in the management of lung cancer.	2003	To study the impact of age on the survival of patients with lung cancer.	A quantitative study with people admitted to 48 hospitals in the United Kingdom.	English
( 20 )	Williams D, Bennett K, Feely J.	Evidence for an age and gender bias in the secondary prevention of ischaemic heart disease in primary care.	2003	To determine whether there is gender or age bias in prescribing important secondary preventive therapies for ischemic heart disease in primary care.	A quantitative study of patients with ischemic heart disease in the East Region of the General Medical Services scheme from Ireland.	English
( 21 )	Bhalla A, Grieve R, Tilling K, Rudd AG, Wolfe CD.	Older stroke patients in Europe: stroke care and determinants of outcome.	2004	To estimate the care structure and process. To identify independent factors associated with three-month mortality and functional outcomes in patients aged over 75 years old.	A quantitative study carried out with stroke victims in 13 hospitals from 10 European countries.	English
( 22 )	Van Delden JJ, Vrakking AM, Van Der Heide A, Van Der Maas PJ.	Medical decision making in scarcity situations.	2004	To explore the clinicians’ views on several topics that have emerged in the debate over the allocation of scarce resources with a particular focus on the role of age and to compare the, with the policymakers’ views.	A qualitative study with oncologists, cardiologists and nursing-home physicians in the Southwest region of the Netherlands.	English
( 23 )	Gunderson A, Tomkowiak J, Menachemi N, Brooks R.	Rural physicians’ attitudes toward the elderly: evidence of ageism?	2005	To examine the perceptions and attitudes of rural Florida physicians who routinely provide care to older adults.	A qualitative study carried out with physicians who care for aged people living in rural areas in Florida, United States.	English
( 24 )	Finger RP, Ali M, Earnest J, Nirmalan PK.	Cataract surgery in Andhra Pradesh state, India: an investigation into uptake following outreach screening camps*.*	2007	To analyze the decision-making processes that lead to accepting cataract surgery services offered during outreach screening camps, investigated in people with cataracts in India.	An exploratory and qualitative study with cataract patients from two eye hospitals in Hyderabad, India.	English
( 25 )	Manthorpe J, Clough R, Cornes M, Bright L, Moriarty J, Iliffe S.	Four years on: the impact of the National Service Framework for Older People on the experiences, expectations and views of older people.	2007	To evaluate the impact of the National Framework of Services for Older Adults on aged people’s experiences and expectations.	A qualitative study carried out with aged people and their caregivers who use health services in England.	English
( 26 )	Pedersen R, Nortvedt P, Nordhaug M, Slettebø A, Grøthe KH, Kirkevold M, et al.	In quest of justice? Clinical prioritisation in healthcare for the aged.	2008	To explore which criteria, values and other relevant considerations are important in clinical priorities in health services for aged patients.	A qualitative study with physicians and nurses who care for aged people in public hospitals and nursing homes in different parts of Norway.	English
( 27 )	Hudelson P, Kolly V, Perneger T.	Patients’ perceptions of discrimination during hospitalization.	2010	To identify sources of perceived discrimination during hospitalization and to examine the relationship of perceived discrimination with patient and hospital characteristics, and with patient care ratings.	A quantitative study with adults assisted in hospitals belonging to the University of Geneva, Switzerland.	English
( 28 )	Mitford E, Reay R, McCabe K, Paxton R, Turkington D.	Ageism in first episode psychosis.	2010	To compare the incidence, diagnosis groups and hospitalization of two different age groups with first-episode psychosis.	A quantitative study using the PACE (Population-Adjusted Clinical Epidemiology) database in Northumberland, United Kingdom.	English
( 29 )	Protière C, Viens P, Rousseau F, Moatti JP.	Prescribers’ attitudes toward elderly breast cancer patients. Discrimination or empathy?	2010	To identify relevant characteristics of physicians, such as their sociodemographic characteristics, experience, knowledge of geriatric testing, “practice style effects” and belief in the effectiveness of the various adjuvant treatments available.	A quantitative study with oncologists and radiotherapists who treat people with breast cancer, in hospitals from France.	English
( 30 )	Clarke LH, Bennett EV, Korotchenko A.	Negotiating vulnerabilities: how older adults with multiple chronic conditions interact with physicians.	2014	To understand how aged people perceived and experienced the care provided by their primary care physicians.	A qualitative study with women and men with chronic diseases who use health services in Canada.	English
( 31 )	Forrest L, Adams J, White M, Rubin G.	Factors associated with timeliness of post-primary care referral, diagnosis and treatment for lung cancer: population-based, data-linkage study.	2014	To investigate the factors (socioeconomic status, age, gender, histology, comorbidity, year of diagnosis, stage and performance status) that may influence the likelihood of referral to post-primary care, diagnosis and treatment within the target times.	A quantitative study with people diagnosed with lung cancer, obtained from the Northern and Yorkshire Cancer Registry and Information Service, in England.	English
( 32 )	Polat U, Karadaǧ A, Ulger Z, Demir N.	Nurses’ and physicians’ perceptions of older people and attitudes towards older people: Ageism in a hospital in Turkey.	2014	To determine the perceptions of old age and the prevalence of aging among nurses and physicians.	A quantitative study with nurses and physicians in the medical and surgical clinics of a university hospital from Turkey.	English
( 33 )	Skirbekk H, Nortvedt P.	Inadequate treatment for elderly patients: professional norms and tight budgets could cause “ageism” in hospitals.	2014	To analyze ethical care considerations among health professionals when treating and prioritizing aged patients in Norway.	A qualitative study with physicians and nurses from general hospitals in Norway.	English
( 34 )	Ben-Harush A, Shiovitz-Ezra S, Doron I, Alon S, Leibovitz A, Golander H, et al.	Ageism among physicians, nurses, and social workers: findings from a qualitative study.	2016	To investigate ageism among health professionals in therapeutic settings in Israel.	A qualitative study with 76 nurses specialized in Oncology, at a specialized center from in Israel.	English
( 35 )	Davis T, Teaster PB, Thornton A, Watkins JF, Alexander L, Zanjani F.	Primary Care Providers’ HIV Prevention Practices Among Older Adults.	2016	To understand the HIV* prevention practices of providers and primary care among older adults. To understand the factors that affect provision of HIV education materials to older adults and HIV screening practices by the providers.	A qualitative study carried out with physicians and nurses who care for patients over 50 years of age in primary health care, in the state of Kentucky, United States.	English
( 36 )	Schroyen S, Missotten P, Jerusalem G, Gilles C, Adam S.	Ageism and caring attitudes among nurses in oncology.	2016	To replicate the results of previous studies that reported differential support of medical treatment according to patient age in a different population of health professionals (nurses instead of physicians). To determine whether support for expensive immunotherapy, adjuvant chemotherapy, or breast reconstruction is related to age bias among nurses.	A qualitative study carried out with nurses specialized in Oncology and working in different units of the Sart Tilman Liège University Hospital, Belgium.	English
( 37 )	Taverner T, Baumbusch J, Taipale P.	Normalization of Neglect: A Grounded Theory of RNs’ Experiences as Family Caregivers of Hospitalized Seniors.	2016	To develop a theory about the Nursing care performance, as described by caregivers of aged people in the intensive care sector.	A qualitative study with aged women and caregivers recruited from two large hospitals in British Columbia.	English
( 38 )	Demetriadou E, Kokkinou M, Metaxas G, Kyriakides E, Kyprianou T.	Psychological support for families of ICU patients: longitudinal documentation of the service.	2017	To evaluate and describe the need for psychological support for relatives of patients admitted to the ICU†, for the period between 2011 and 2014. To identify the health professionals’ perception regarding the need for psychological support.	A qualitative study carried out based on the documentation of psychological assistance to relatives of patients admitted to the ICU from 2011 to 2014 in Cyprus.	English
( 39 )	Di Rosa M, Chiatti C, Rimland JM, Capasso M, Scandali VM, Prospero E, et al.	Ageism and surgical treatment of breast cancer in Italian hospitals.	2018	To assess whether the patient’s age is a factor that influences the type of surgical treatment for breast cancer in Italy. To assess the existence and characteristics of possible regional differences in the type of surgical treatment for breast cancer.	A retrospective study based on national hospital discharge records, in Italy.	English
( 40 )	Forti P, Maioli F, Magni E, Ragazzoni L, Piperno R, Zoli M, et al.	Risk of Exclusion From Stroke Rehabilitation in the Oldest Old.	2018	To investigate whether older age ≥ 85 (years old) is an independent predictor of exclusion from stroke rehabilitation.	A retrospective cohort study carried out with aged people diagnosed with stroke, in an Italian hospital.	English
( 41 )	Kiplagat J, Huschke S.	HIV testing and counselling experiences: a qualitative study of older adults living with HIV in western Kenya.	2018	It describes HIV testing experiences of older adults living with HIV, and how their age shaped their interaction and treatment received during HIV testing and diagnosis.	A qualitative study conducted with HIV-infected people aged ≥ 50 years old using rural and urban clinic services in western Kenya.	English
( 42 )	Schatz, E, Seeley, J, Negin, J, Mugisha J.	They ‘don’t cure old age’: Older Ugandans’ delays to health-care access.	2018	To examine the factors that cause older Ugandans to delay accessing health care.	A qualitative study with aged people in the Kalungu district, rural southwestern Uganda.	English
( 43 )	Schroyen S, Adam S, Marquet M, Jerusalem G, Thiel S, Giraudet AL, et al.	Communication of healthcare professionals: Is there ageism?	2018	To observe whether characteristics of older adults’ speech (positive or negative) are more frequent in professionals with a negative view of aging.	A qualitative study with physicians from the city of Liège, Belgium.	English
( 44 )	Wiel E, Di Pompéo C, Segal N, Luc G, Marc JB, Vanderstraeten C, et al.	Age discrimination in out-of-hospital cardiac arrest care: a case-control study.	2018	To compare out-of-hospital cardiac arrest care and outcomes among young patients.	A quantitative study with data from adults with cancer, extracted from the French national cardiac arrest registry, in France.	English
( 45 )	Dobrowolska B, Jędrzejkiewicz B, Pilewska-Kozak A, Zarzycka D, Ślusarska B, Deluga A, et al.	Age discrimination in healthcare institutions perceived by seniors and students.	2019	To explore age-based discrimination in health institutions perceived by older adults and medical and nursing students.	A multi-method study with 65-year-olds attending the University of Old Age in eastern Poland.	English
( 46 )	Shin DW, Park K, Jeong A, Yang HK, Kim SY, Cho M, et al.	Experience with age discrimination and attitudes toward ageism in older patients with cancer and their caregivers: A nationwide Korean survey.	2019	To understand the view of aged patients themselves in relation to their care in terms of their relationship with age. To determine: 1) Whether older adults and their caregivers believe that aged patients should be provided the same care level as younger cancer patients; 2) Whether aged patients experienced any age-based discrimination during their cancer treatment.	A quantitative research study conducted with aged people with cancer and their caregivers who participated in the National Cancer Center and another ten regional cancer centers in Korea.	English
( 47 )	Heyman N, Osman I, Ben Natan M.	Ageist attitudes among healthcare professionals and older patients in a geriatric rehabilitation facility and their association with patients’ satisfaction with care.	2020	To explore the prevalence of age attitudes (based on the specific age group) in older adults admitted to geriatric rehabilitation, as well as the association between satisfaction with care and age attitudes. To estimate whether there is an association between patient satisfaction with care and age attitudes among health professionals working in the rehabilitation unit. To explore the prevalence of age attitudes among health professionals.	A quantitative study with health professionals and aged people, in a rehabilitation department of a geriatric medical center located in Mid-North Israel.	English
( 48 )	Kessler EM, Blachetta C.	Age cues in patients’ descriptions influence treatment attitudes.	2020	To investigate how age cues in the psychotherapeutic context influence health professionals’ treatment attitudes.	A quantitative study with psychotherapists from the Behavioral Therapy Association in Progress, Germany.	English
( 49 )	Lee J, Yu H, Cho HH, Kim M, Yang S.	Ageism between Medical and Preliminary Medical Persons in Korea.	2020	To analyze trends in ageism among health professionals and medical students in the Republic of Korea.	A quantitative study with health professionals and aged people, conducted in the Republic of Korea.	English
( 50 )	Motsohi T, Namane M, Anele AC, Abbas M, Kalula SZ.	Older persons’ experience with health care at two primary level clinics in Cape Town, South Africa: a qualitative assessment.	2020	To evaluate how older adults experience health care provision in two primary care clinics; and to identify perceived gaps in health care for aged people.	A qualitative study with aged people in two primary care units from the suburbs of Cape Town, South Africa.	English
( 51 )	Rababa M, Hammouri AM, Hweidi IM, Ellis JL.	Association of Nurses’ Level of Knowledge and Attitudes to Ageism Toward Older Adults: Cross-sectional Study.	2020	To examine how the sociodemographic and professional characteristics of nurses in Jordan correlate with their levels of knowledge, attitudes and age in relation to older adults.	A descriptive, correlational and cross-sectional study carried out with nurses who work at a public hospital and a university hospital in Irbid, northern Jordan.	English
( 52 )	Tomioka S, Rosenberg M, Fushimi K, Matsuda S.	An analysis of equity in treatment of hip fractures for older patients with dementia in acute care hospitals: observational study using nationwide hospital claims data in Japan.	2020	To assess whether dementia status is associated with poorer care by examining the association of the patient’s dementia status with the likelihood of undergoing surgery and waiting time until hip fracture surgery in acute care hospitals in Japan.	A quantitative study with victims of closed hip fracture, whose data were extracted from the *Diagnosis Procedure Combination* database in Japan.	English
( 53 )	Hwang EH, Kim KH.	Quality of Gerontological Nursing and Ageism: What Factors Influence on Nurses’ Ageism in South Korea?	2021	To identify the factors that influence nurses’ ageist attitudes	A quantitative study with nurses from two general hospitals in a provincial town from South Korea.	English

*HIV = Human Immunodeficiency Virus; †ICU = Intensive Care Unit

The time dimension of the studies found was mostly understood between 2000 and 2020; prior to this period, an article published in 1985 and another in 1996 were identified. In relation to the types of study, 59% were quantitative, 35% qualitative and 4% mixed-methods. The participants surveyed were mostly aged people who use health services (57%), followed by health professionals (40%) and caregivers of older adults (3%). The contexts were mostly public and private hospitals in different countries, and a minority in primary health care contexts. The ageism expressions are shown in Figure 4.

**Figure 4 -:** Mapping of ageism expressions directed to older adults. Feira de Santana, BA, Brazil, 2021

Ageism expressions directed to older adults in health services
**Less diagnostic research** Unquestioned sexual history in the psychiatric evaluation of aged men( 16 ). Reduced histological verification rates( 17 ). Brief anamnesis( 18 ). Inadequate diagnostic investigations( 21 ).
**Restricted access to treatments** Fewer curative surgeries and chemotherapy( 17 ). Lower probability to receive statin, b-blocker or aspirin and other potentially beneficial treatments( 20 ^,^ 29 ). Inadequate treatment for sexual dysfunction( 21 ). Lower probability to receive timely cancer treatment. Unjustified delays( 31 ). Omission of information about disease status and treatment options( 46 ^,^ 49 ).
**Reduction of surgical interventions** Lower probability to receive treatment (surgery, radiotherapy, chemotherapy) of any kind( 19 ). Lower probability to access resources made available for cataract surgery( 24 ). Lower probability of breast reconstruction and less incentive to undergo immunotherapy( 36 ). Few conservative surgeries, regardless of the clinical severity of breast cancer( 39 ). People aged between 80 and 90 years old are less likely to be operated on when compared to patients aged between 65 and 79 years old( 52 ).
**Use of inappropriate language** Condescending and childish language( 34 ^,^ 37 ). Aged people were ignored and shunned, and privacy was not guaranteed( 37 ). Instructions in inaccessible language, inhibiting older adults’ participation( 41 ). Very short statements that express negative characteristics( 43 ).
**Low access to health services and resources** Lower probability to be screened to an Emergency Department resuscitation room( 13 ). Health professionals less willing to treat aged people with suicidal ideation( 14 ). Limited access to organs for transplantation due to age( 22 ). Aged people experiencing psychosis without access to relevant services, guidelines and funding( 28 ). Low probability of accessing rehabilitation services( 34 ^,^ 40 ).
**Inadequate care** Aged people feel that they do not access the same care, resources and guidance as younger individuals( 16 ^,^ 18 ^,^ 27 ^,^ 46 ). Suicide risk is not assessed( 18 ). Inattention to older adults’ needs( 26 ). Perception of incomplete, uncomprehending medical care and discriminatory practices during interactions between physicians and aged people( 30 ). Aged people with no cure possibility through medical treatment are also less prioritized in basic Nursing care( 33 ). Rushed service during clinical consultation( 41 ). Aged people receive less intensive care during cardiac arrest when compared to younger groups( 44 ). Lack of investment in care and lack of consideration related to the holistic needs and contextual challenges faced by older adults( 50 ).
**Inattention to family members** Aged people’s relatives receive less psychological support, regardless of prognosis( 38 ).
**Anti-aging behaviors** Negative and anti-aging attitudes on the part of employees towards old age( 25 ). Social devaluation( 30 ). Embarrassment in caring for aged men with a sexually transmitted infection( 41 ). Aged men, older adults and people with lower schooling levels felt discriminated against because of their age in clinics and hospitals( 45 ). Jewish health professionals showed more anti-aging attitudes than Arabs, even those who specialize in the field( 47 ). Older nurses reported high frequency of negative age-related behaviors in everyday life( 51 ). General hospital nurses have anxiety and fear of old age( 53 ).
**Beliefs and stereotypes** Health professionals are more likely to feel that suicidal ideation in older adults is normal and not to use therapeutic strategies( 14 ). Rural physicians have a negative perception of people over 85 years old, regardless of all the information about health status and functional and cognitive capacity( 23 ). Perception of aged people as weak and sick, with diminished mental abilities, intolerant and inflexible, disabled and difficult to adhere to treatments( 32 ). Beliefs that aged people are less sexually active and are uncomfortable talking about sex( 35 ). Negative affect towards aged people, less interest in treating them, perception that treatments are not successful and have worse prognosis( 48 ).
**Violent approach** Aged people were treated as objects and responsibility for their care was transferred to the family( 37 ). Health professionals yelled at, were rude to and accused aged people of wasting their time and taking medications that should be intended for younger individuals; they criticized for not listening and understanding the guidelines, not following the protocols and considering the illegitimate complaints; older adults were disrespected, devalued and labeled as uncooperative and unintelligent( 42 ).

The coping measures against ageism directed to older adults are shown in Figure 5.

**Figure 5 -:** Mapping of the coping measures against ageism directed to older adults in health services. Feira de Santana, BA, Brazil, 2021

Coping measures against ageism directed to older adults in health services
Improve communication between aged people and medical team( 16 ). Invest in and carry out scientific research studies( 15 ^,^ 23 ^,^ 31 - 32 ^,^ 51 ). Train and update scientific knowledge for health professionals( 14 ^,^ 30 ^,^ 35 - 36 ^,^ 38 ^,^ 41 ^,^ 45 ^,^ 47 - 48 ^,^ 53 ). Listen to citizens and hospital administrators and their perspectives on older adults’ health priorities( 26 ). Stimulate the work of physicians and nurses in the Geriatrics and Gerontology areas( 32 ). Educate family caregivers, aged people and health professionals to fight against ageism and make decisions based on scientific evidence( 46 ). Conduct campaigns to raise awareness of the aging process and old age( 50 - 51 ).

## Discussion

This study mapped the scientific literature on the ageism expressions in health services and showed that, when using these services, aged people are the target of less diagnostic research, have restricted access to different types of treatments and surgical interventions, and also receive inadequate care in general, manifested through inappropriate language by health professionals, anti-aging behaviors and care based on beliefs and stereotypes, even reaching violent approaches. Even in the case of aged people’s relatives, they receive less psychological support, connoting age bias. The coping mechanisms mainly consist of educational interventions and strengthening of communication channels between aged people, health professionals and institutional managers.

Attention to ageism has as its international landmark the adoption of the Madrid International Action Plan on Aging, dated 2002, which has guided public civil and health actions in various parts of the planet. However, only in May 2016, the 194 member states of the World Health Organization, in cooperation with other partners, carried out the Global Campaign to Combat Ageism to improve older adults’ everyday life and optimize the implementation of public policies to confer visibility to the phenomenon in the search for change in the way people think, feel and act when it comes to age and aging( 54 ).

Added to this social emphasis landmark is the countries’ commitment to deal with ageism, reasserted in the United Nations Decade for Healthy Aging (2021–2030) on December 14^th^, 2020, based on the 10-year action plan: Global strategy/action plan on aging and health (2016–2030); 2030 Agenda for Sustainable Development. Both were endorsed in August 2020 by the World Health Assembly and in December 2020 by the United Nations General Assembly( 55 ). Thus, prevention and confrontation of ageism has become one of the United Nations’ four priority action areas. This movement has been reflected on scientific productions, where an increase in the development of research studies can be observed from 2001 onwards.

The theme of aging in contemporary times has generated intellectual and political concerns in capitalist societies, not only because it constitutes a demographic phenomenon but also because it involves economic, social, political, cultural and ethical aspects of significant commotion, which determine the quality of societies’ commitment to human rights. In this context, current dualities can be seen: if on the one hand human/population aging represents a breakthrough in medicine/public health — combined with a culture of respect for differences guaranteed by rights and public policies, on the other hand, the increase in the number of aged individuals in society is seen as an impediment to economic growth — a paradox with opposing logics of profit *versus* human needs( 56 ).

In this sense, the Neoliberal logic that devalues the most aged maintains the propagation of negative images and attitudes about older adults, a scenario that is transposed into health professionals’ daily work in services, through the development of stereotypes (thoughts), prejudices (feelings) and discrimination (action), which are expressed at the interpersonal and institutional levels and permeate health professionals’ work process^(7,57)^.

Interpersonal ageism comprises prejudiced attitudes towards the aging process, including self-directed ageism( 1 ). This was evidenced in the results of this study based on the expressions manifested in the way health professionals act during care provision through anti-aging behaviors based on beliefs and stereotypes that affect the quality of the health care provided to older adults, such as: brief anamnesis( 21 ^,^ 24 ); fewer guidelines on treatments or guidance with language that is not understandable or childish( 34 ); embarrassment when caring for aged people( 35 ^,^ 41 ); transfer of the care responsibility to the family( 37 ); unconcern with lack of privacy, and unequal, inhumane, disrespectful treatment that disregards the specificities and vulnerabilities of old age( 37 ^,^ 42 ); and negative perception about the treatment and about the aged person as a whole( 23 ^,^ 25 ^,^ 32 ^,^ 47 - 48 ^,^ 51 ).

Family relationships are decisive for interpersonal ageism( 5 ), a context in which the function of cultivating beliefs, values and principles that constitute the culture of a group is exercised, at the same time, in which younger people respond to the demands of liquid society. The adjective “liquid” gathers characteristics of contemporary individualistic society, with little solidarity, weak community ties, competitive, focused on the speed to perform tasks and on indiscriminate consumption of products, a scenario widely publicized on social networks and communication channels( 57 ).

As a result, we experience an exclusionary society with frequent intergenerational conflicts, whose stigmatization takes place in family interactions through attitudes of discredit, contempt and use of derogatory adjectives, assimilated by older adults, and which conform to self-image and a deteriorated identity. Therefore, separation or abandonment by the family is common, especially in situations of physical difficulties that require greater attention and protective care( 58 ).

All these ageism expressions, mapped by this review, reveal that older adults are considered a burden for society and held responsible for the increase in the public budget disputed both by labor and by capital. Despite all the ageism expressions, the aged population will still suffer from the highest rates of public and private neglect, as well as social discrimination, poverty and violence( 59 ).

Although the objective reality of population aging results from improvements in access to technological and health resources, the subjective reality of age stereotypes moves in a negative direction, which promotes inequalities, illness and exclusion, and which can be partially explained by the multibillion-dollar anti-aging industry that promotes stigmatization by placing supposed attributes of the old into a category that must be fought against and that should be avoided at all costs( 60 ). This is reflected in the assistance received in the health services, when the professionals, due to having negative views of old age and aging, use these perceptions to provide care, even unconsciously.

Although directly related to interpersonal ageism, institutional ageism differs from it by involving the inclusion of age principles in formal rules and procedures and in broader institutional cultures( 7 ), which does not necessarily require intention or awareness of bias against older adults, as the existence of such institutional prejudice is frequently not recognized and the institution’s rules, norms and practices are longstanding, turning it into a phenomenon of social coercion. The consequence of the latter is natural acceptance, in which there is hardly room to criticize, disturb or modify, resulting in implicit and explicit effects of this phenomenon( 61 ), as impacts on the physical and mental health, social well-being and economy of older people, families and society( 62 ). In addition, it is possible that institutional ageism favors labeling people according to their age, depersonalizing them and disregarding their subjectivity and specificities.

The ageism expressions presented in this review denote how devalued aged people can be when accessing health services and how chronological age can undervalue the assistance provided to them, while creating barriers in accessing health resources such as surgical interventions( 17 ^,^ 24 ^,^ 36 ^,^ 39 ^,^ 52 ), rehabilitation services( 21 ^,^ 40 ), drug prescriptions( 20 ^,^ 29 ) and various treatments( 13 ^,^ 15 ^,^ 19 ^,^ 38 ^,^ 46 ), lack of assistance, unjustified delays, less effort in care actions for this population group and care prioritization for younger people. Other studies confirm the deleterious consequences of ageism on older adults’ health( 3 - 5 ).

The repercussions of ageism are also reflected on the nature of the care received by aged people’s relatives, who tend to receive less psychological support regardless of the clinical outcome( 38 ), and which can impose new and intense challenges when it comes to older adults with some dependence degree or who need long-term care. This happens because the absence of a network to protect and defend rights leads aged people to face serious problems in their everyday lives and the most burdened end up being family members of care-dependent older adults, in particular( 56 ).

In health and long-term care institutions, ageism is ubiquitous, socially accepted, mostly undetected and heavily institutionalized; it is extremely detrimental to health and well-being, associated with poorer performance in physical and cognitive tasks, poorer physical and mental health, slower recovery from disability and decreased longevity; it also influences social values and shapes the research and politics focus, including how problems are conceptualized, the solutions proposed and how institutions develop and implement rules and procedures( 62 ).

In view of this, it becomes necessary that coping strategies are permanent and continuous, and that they are implemented top-down (from society to the individual) and bottom-up (from the individual to society, considering the role of aged people), with the joint objectives of reinforcing conditions that promote positive age images and mitigating conditions that promote negative age stereotypes( 60 ). This is because the evidence shows that higher knowledge levels about aging are associated with fewer anti-aging attitudes( 47 ).

During the training processes, future health professionals are trained to treat and achieve results through hospitalization, timely treatment of diseases, and considering age as an isolated health/disease marker( 45 ). Although many needs of aged people can be met by interdisciplinary care in the Primary Health Care context, the tenuous linkage of actions to the social determinants in health shows professionals’ difficulties to move away from biomedical care, which is purely repetitive, towards comprehensive and interactionist care, reiterating the hospital perspective, specialized and circumscribed to the disease, which strengthens historical and hegemonic paradigms in Brazil( 63 ): old age is a life stage in which the subjects’ development is completed, and that losses and frustrations prevail in the face of physical decline, despite having experience and wisdom( 64 ), reinforcing negative connotations and reflecting pejorative stereotypes that establish disease as an intrinsic condition of old age( 45 ).

Therefore, it becomes necessary to analyze age in the context of physical functioning, associated diseases, life expectancy, cognitive capacity, functional independence and nutritional status, among other important indicators that support the recommendation of appropriate treatments( 49 ). More reflective approaches, focusing on aged people’s needs and on the integration of the age variable in analyses of the human being’s life cycle, in the approach to the physiological aspects of aging and in expanded geriatric-gerontological assessments( 46 ), are essential for centralized care in health promotion and in the prevention of risks and health problems, considering older adults and their social, family, economic and cultural contexts.

It is also necessary to recognize the presence of ageism in health environments( 45 ) so that health professionals become self-monitoring regarding practices guided by stereotypes, prejudices and age discrimination and that the management of health services encourage and implement educational interventions among health professionals to raise awareness and sensitize them about fighting against ageism in health services.

Another struggle front evidenced in the results of this study is related to the funding of age-related research studies and projects, to the creation of educational campaigns for public awareness about age, aging, old age and ageism( 51 ), and to the dissemination of aged people’s rights and duties to empower and stimulate emancipating strategies in communities, churches, social groups, local councils for older adults’ rights, universities open to aged people, neighborhood associations and collectives.

Ageism is a topic little known by the population in general, and the results of this review contribute not only to raising the discussion on the subject matter but also to advance scientific knowledge and innovate, by showing how age-related stereotypes, prejudices and discrimination are expressed in the health services’ routines, revealing how unequal and unfair the assistance received by aged people can be in the contexts where they should be cared for.

The limitations of this study consist in the identification of the ageism expressions in health services explicitly reported by the authors of the articles analyzed, in addition to the fact that some reviewers did not obtain full texts, even after requesting them from the authors.

## Conclusion

Ageism directed at older people in health services is manifested by negative expressions of discrimination, prejudice and age stereotypes that restrict aged people and their families in accessing health resources and services, minimizing opportunities for treatment, rehabilitation and cure, which pervade the work process in health. The coping strategies involve educational interventions through health education, awareness-raising campaigns, updating scientific knowledge and expanding communication channels between aged people, health professionals and managers. The ageism expressions in the context of health services that make up the Unified Health System constitute a knowledge gap, as well as the implicit ageism expressions.

The studies mapped deal with explicit ageism, in which there is knowledge of its existence based on primary studies identified; therefore, there seems to be a gap in the scientific knowledge about the forms of stereotypes, prejudices and discrimination that implicitly coexist in health services. Similarly, the absence of studies in the Brazilian reality limits the authors’ inferences and precludes performing analyses in the context of the health services that make up the Unified Health System.

Understanding these expressions may generate implications for a professional practice that recognizes the presence of interpersonal or institutional ageism, making professionals vigilant for care/neglect guided by age bias and sensitive to the implementation of coping strategies. With regard to the academic environment, the findings of this study may guide research studies that analyze ageism in the Brazilian reality by listening to aged people, family members, health professionals and managers in various public and private health services, as well as by grounding extension actions to face and prevent ageism in health contexts.
